# Hereditary thrombophilia parameters in children with autism spectrum disorder and their mothers

**DOI:** 10.3389/fped.2025.1680354

**Published:** 2025-12-11

**Authors:** Perihan Cam Ray, Merve Doğan, Sevcan Bozdoğan, Gonca Gül, Hülya Binokay

**Affiliations:** 1 Department of Child and Adolescent Psychiatry, Cukurova University Faculty of Medicine, Adana, Türkiye; 2 Department of Medical Genetics, Cukurova University Faculty of Medicine, Adana, Türkiye; 3 Department of Biostatistics, Cukurova University Faculty of Medicine, Adana, Türkiye

**Keywords:** autism spectrum disorder, hereditary thrombophilia, factor XIII Va34Leu, neurodevelopment, genetic polymorphism, MTHFR gene mutation

## Abstract

**Objective:**

Autism Spectrum Disorder (ASD) is a complex neurodevelopmental condition influencyed by genetic and environmental factors. There is emerging evidence of an association between hereditary thrombophilia and ASD, potentially mediated by impaired placental perfusion and resultant neuroinflammatory processes. This study aimed to investigate the frequency of thrombophilia-related genetic polymorphisms in children diagnosed with ASD and their mothers.

**Methods:**

A total of 24 children with ASD aged 2-6 and their mothers were compared with 24 age-matched healthy children and their mothers. Sociodemographic, developmental and genetic data were collected. A psychiatric evaluation was performed according to the fifth edition of the Diagnostic and Statistical Manual of Mental Disorders (DSM-5), and the children were assessed using the Autism Behavior Checklist (ABC), the Modified Checklist for Autism in Toddlers (M-CHAT) and the Ankara Developmental Screening Inventory (ADSI). Thrombophilia-related polymorphisms, including FVL G1691A, FII G20210A, C677T MTHFR and 1298AC MTHFR, FXIII-Val34Leu and PAI-1 4G/5G, were analyzed using PCR-based methods. Statistical comparisons and logistic regression analyses were performed to evaluate associations with ASD.

**Results:**

The FXIII-Val34Leu heterozygous variant was significantly more prevalent in children with ASD (37.5% vs. 8.3%, *p* = 0.036) and their mothers (54.2% vs. 16.7%, *p* = 0.015) than in the control group. Logistic regression analysis revealed that the presence of the FXIII-Val34Leu heterozygous polymorphism in either the mother or child was associated with an approximately 4.130-fold increase in the odds of ASD (adjusted odds ratio = 4.130, 95% confidence interval = 1.180–5.300, *p* = 0.027). Other thrombophilia polymorphisms did not differ significantly between groups. Additionally, children with ASD exhibited significant delays in speech development and lower developmental scores across several domains.

**Conclusion:**

This study is among the first to examine the FXIII Val34Leu mutation in children with ASD and their mothers. Further large-scale, longitudinal studies are needed to investigate thrombophilia markers in relation to ASD.

## Introduction

Autism Spectrum Disorder (ASD) is a neurodevelopmental condition characterized by deficits in social communication and interaction, as well as restricted and repetitive patterns of behavior, emerging in early childhood (1). The etiopathogenesis of ASD is complex and believed to result from the interaction of genetic, epigenetic, and environmental factors (2). Genomic-level research has identified numerous genetic variants that influence synaptic function, neuronal migration, and neuroinflammation. These variants may disrupt neurodevelopmental processes by affecting the development of the cerebral cortex, particularly during the prenatal period (3). Additionally, mechanisms critical to nervous system development—synaptic plasticity, cytoskeletal organization, and blood–brain barrier integrity—have been implicated in the pathogenesis of ASD.

In recent years, increasing evidence suggests that ASD may be associated not only with neuronal mechanisms but also with vascular and hematologic pathways. In particular, alterations in cerebral blood flow, abnormal vascular development, and coagulation system dysfunctions have been proposed to be associated with ASD (4). Hereditary thrombophilia, which increases the tendency for thrombosis in both the mother and fetus, may indirectly influence brain development during the intrauterine period and thereby elevate the risk of ASD (5). Microthrombi resulting from thrombophilia may trigger neuroinflammation and exert adverse effects on neurodevelopmental outcomes.

Hereditary thrombophilia is a condition that arises due to mutations causing a genetic predisposition to venous thromboembolism (6). Polymorphisms in genes such as 5,10-methylenetetrahydrofolate reductase (MTHFR), Factor V Leiden (FVL), factor II prothrombin (FII), Factor XIII (FXIII) Val34Leu, and plasminogen activator inhibitor-1 (PAI-1) are known to contribute to this condition (7). It has been suggested that genetic coagulation disorders in pregnant women may impair placental circulation by disrupting uteroplacental perfusion, potentially leading to adverse effects on fetal neurodevelopment (8).

The aim of this study is to investigate whether there is a relationship between ASD and hereditary thrombophilia by examining thrombophilia parameters in children diagnosed with ASD and their mothers. In addition, the study aims to explore whether these parameters could serve as potential screening biomarkers. The lack of comprehensive studies in the literature that simultaneously assess both maternal and child thrombophilia profiles highlights the potential significance and contribution of this research to the existing body of knowledge.

## Materials and methods

### Sample

This study included 24 children (5 girls, 19 boys), aged 2–6 years who were diagnosed with ASD according to DSM-5 criteria, and their mothers (9). These participants were recruited from the Child and Adolescent Psychiatry Outpatient Clinic at Çukurova University Faculty of Medicine. An additional 24 age-matched healthy children (10 girls, 14 boys) and their mothers were enrolled as the control group. Written informed consent was obtained from all parents after detailed information about the study's purpose and procedures was provided. The researchers (M.D. and P.Ç.R.) collected data and performed evaluations through face-to-face interviews. The study was conducted in accordance with the principles of the Declaration of Helsinki and was approved by the Çukurova University Non-Interventional Clinical Research Ethics Committee (April 2, 2021; Approval no.: 110).

### Procedure

#### Assessment of the patient group

The ASD group comprises children aged 2–6 who have been diagnosed with ASD and assessed functioning class according to DSM-5 criteria (9). At the clinic, patients are evaluated by research assistants, pediatric psychiatrists, and faculty members. The research assistants assess the patients and then consult with the faculty members to create a diagnosis and treatment plan. During the evaluation, forms are used to collect sociodemographic information and diagnostic criteria from the DSM-5, as well as standard psychometric measurements, to support diagnoses. The Modified Checklist for Autism in Toddlers (M-CHAT), the Autism Behavior Checklist (ABC) and the Ankara Developmental Screening Inventory (ADSI) are routinely administered to children under six who are referred to our outpatient clinic with suspected developmental delay. In this study, all participants were evaluated by researchers M.D., P.Ç.R. or G.Ç., with the scales being administered by M.D. However, due to age considerations, the M-CHAT remained descriptive only. Patients received treatment in accordance with clinical guidelines and protocols. The data were thoroughly analyzed, including demographics, prenatal and perinatal history, birth history, other early symptoms, coexisting conditions, and neurodevelopmental and medical data. The parents' demographic data were also analyzed (e.g., age, educational qualifications, and mental and medical conditions). Children with major physical/medical problems, a confirmed diagnosis of a genetic syndrome or inherited metabolic disease, intellectual disability, bipolar disorder, or psychotic disorders were excluded.

#### Assessment of the control group

The group included 24 age-matched children. This group was selected from children who had been assessed by the same team of child and adolescent psychiatrists and were found to have no such disorders. Data from control parents/children were collected in a similar way to that from the patient group. Children with major physical/medical problems, genetic syndromes or inherited metabolic diseases, or mental disorders were excluded.

### The Ankara developmental screening inventory (ADSI)

The ADSI is used in Turkey to assess children's development from 0–6 years. In this study, language, motor, and general development were evaluated using the tool. It was developed for Turkish children by Savasir et al. (1998) (10). It comprises four subscales: communication–language–cognitive, fine motor, gross motor, and social skills–self-care. Administration takes 30–45 min, depending on age and skills. The inventory consists of 154 items, organized by age group, answered by mothers with “Yes,” “No,” or “I don't know.” It is a valid and reliable way of assessing children's development in the Turkish population (10, 11).

### Autism behavior checklist (ABC)

Developed by Krug et al. in 1993, the ABC is a 57-item questionnaire about symptoms of autism (12). Parents/guardians score sensory, relating, body concept and object use, language, social and self-help on a 4-point scale from 0 (no problem) to 3 (severe problem). Total scores range from 0–159, with higher scores indicating greater severity. Irmak et al. (2007) conducted a Turkish study into the scale's validity and reliability (13). They found the correct classification rate of the ABC to be 88%. The cut-off score for the scale was set at 39. The Cronbach's alpha coefficient and the split-half test reliability were found to be 0.92.

### Laboratory and molecular genetic analyses

Peripheral blood samples collected from children and their mothers in EDTA tubes were used for molecular analyses. The polymerase chain reaction (PCR) technique was used to amplify six specific regions to detect polymorphisms in the following genes: FVL G1691A (Factor V Leiden), FII G20210A (prothrombin), C677T, MTHFR A1298C, PAI-1 4G/5G, FXIII Val34Leu polymorphism. The amplified gene products were separated by electrophoresis, and fragment analysis was performed using specialized software to determine the genotypic profiles. Three genotypes of each gene were investigated: normal, heterozygous and variant. In addition, the results of the following tests performed by our outpatient clinic were included: routine vitamin B12 level (pg/mL), vitamin D level (ng/mL) and anti-streptolysin O (ASO) titer (IU/mL).

In the control group, all loci conformed to the expectations of the Hardy–Weinberg principle (exact test: all *p* > 0.05; BH-adjusted *q*-values = 1.00). The smallest *p* value was observed for PAI 4G/5G [*p* = 0.194; minor allele frequency (MAF) = 0.375], whereas the other loci ranged from *p* = 0.683–1.000 (e.g., MTHFR A1298C: *p* = 0.683; FII G20210A: *p* = 1.000; FVL G1691A: *p* = 1.000; MTHFR C677T: *p* = 1.000; FXIII Val34Leu: *p* = 1.000). Control-group MAFs ranged from 0.021–0.396. In the control group of mothers (*n* = 24), all six maternal loci were broadly consistent with Hardy–Weinberg expectations based on exact tests. The smallest *p*-value occurred at the PAI 4G/5G locus (p-exact = 0.024), but this was not significant after Benjamini–Hochberg correction across loci (*q* = 0.146). Exact *p* values for the remaining loci ranged from 0.135–1.000. The minor-allele frequencies (MAF) spanned 0.000–0.354 (FII G20210A = 0.000; FVL G1691A = 0.0625; FXIII Val34Leu = 0.0833; MTHFR C677T = 0.2708; PAI 4G/5G = 0.3333; MTHFR A1298C = 0.3542). FII G20210A was monomorphic among mothers and was therefore uninformative for association testing. Overall, these maternal genotype results suggest that the control sample was adequately genotyped.

### Statistical analysis

Categorical variables were expressed as counts and percentages, whereas continuous variables were summarized as mean ± standard deviation or median (minimum–maximum). Comparisons between the ASD and control groups were performed using chi-square or Fisher's exact test for categorical variables. Categorical comparisons used Pearson's *χ*² test when assumptions were met; when ≥1 expected cell count was <5 (or any observed count was 0), two-sided Fisher's exact test was used. The normality of distribution for continuous variables was confirmed with the Kolmogorov–Smirnov and Shapiro Wilk test. For comparison of continuous variables between two groups, the student's *t* test or Mann–Whitney *U* test was used depending on whether the statistical hypotheses were fulfilled or not. Firth bias-reduced logistic regression analysis was performed to determine significant predictors of autism variable. Multiple group comparisons between the ASD and control groups were conducted using chi-square and *t*-tests, as appropriate. To control the potential increase in false positives due to multiple testing, the Benjamini-Hochberg procedure was applied across the main families of comparisons. Hardy–Weinberg equilibrium (HWE) was evaluated for each maternal locus in the control group using two-sided exact tests, with multiple comparisons controlled by the Benjamini–Hochberg procedure (*q* < 0.05), and monomorphic loci excluded from association analyses. Analyses were performed in R studio 4.2.2 and IBM SPSS Statistics Version 20.0 statistical software package. The statistical level of significance for all tests was considered to be 0.05.

**Figure 1:**
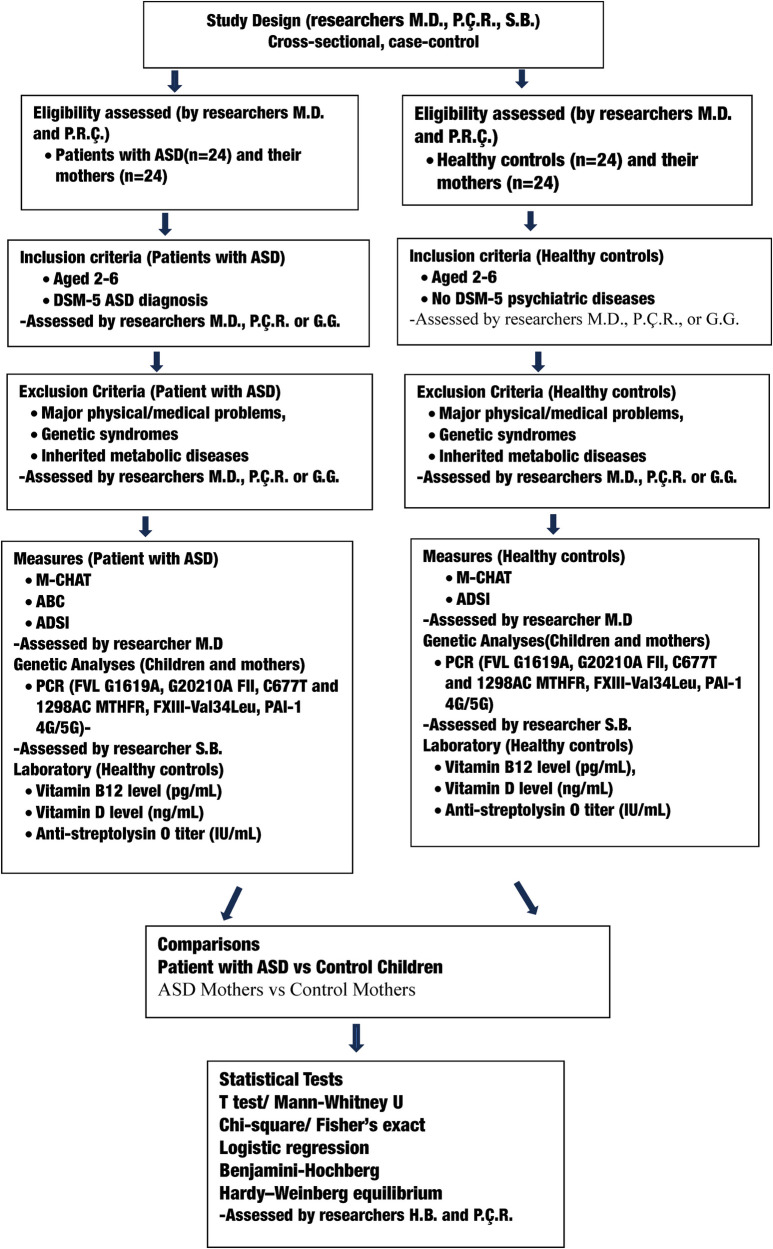
Flow chart of study design, participant selection, measures, comparisons, and statistical analyses. M-CHAT, the modified checklist for autism in toddlers, ABC, autism behavior checklist; ADSI, Ankara development screening inventory; PCR, polymerase chain reaction.

Figure 1 shows a flow chart of the study design.

**Figure 2:**
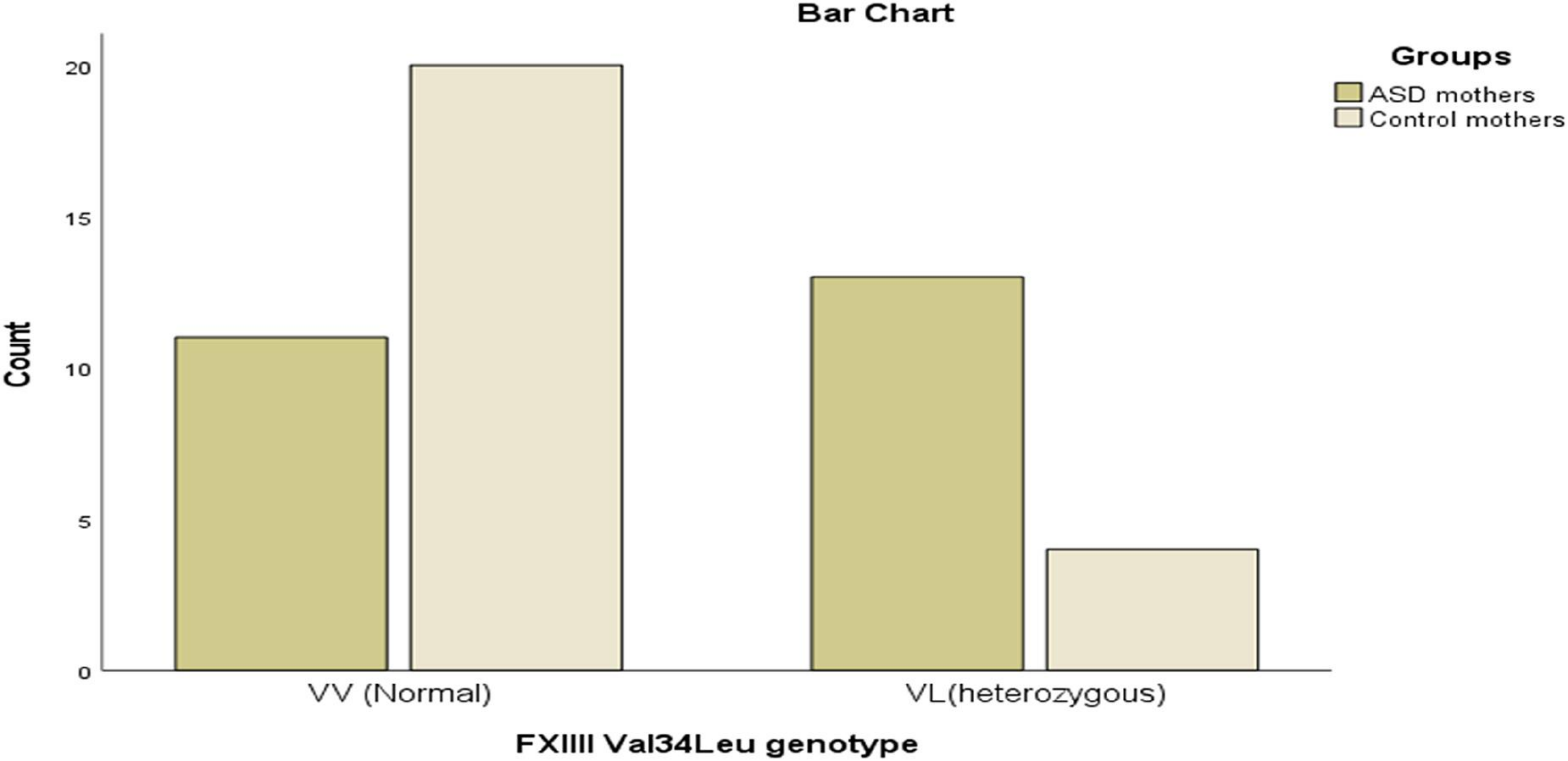
The FXIII-Val34Leu mutation in mothers of children with ASD compared to mothers of healthy children.

## Results

In terms of gender distribution, 79.2% (*n* = 19) of the children in the ASD group were male, whereas the proportion of males in the healthy control group was 58.3% (*n* = 14). Although the groups were not fully matched by sex, this difference was not statistically significant (adjusted *p* = 0.238) (Table 1). Regarding ASD severity level, 6 children (25.0%) had ASD level 1, and 11 (45.8%) had ASD level 2, 7 (29.2%) had ASD level 3. Eleven of the children with ASD (45.8%) were non-verbal. Children with ASD scored lower than healthy children in all domains of the ADSI developmental assessment except the gross motor domain. Articulation disorder was present in 3 patients (12.5%). No statistically significant differences were observed between the groups in terms of paternal or maternal educational level or the presence of medical disease or psychiatric disorder in the parents (all *p* > 0.05) (Table 1).

**Table 1:** Sociodemographic characteristics and comorbidity status of families of children with ASD and healthy controls.

Variable		ASD	Control	*P* values	Adjusted *p*
		(*n* = 24)	(*n* = 24)		
		Counts (%)	Counts (%)		
Sex (child)	Female	5 (20.8)	10 (41.7)	0.119	0.238
	Male	19 (79.2)	14 (58.3)		
Maternal data					
-Educational level	Elementary	13 (54.2)	7 (29.2)	0.101	0.227
	High School	6 (25.0)	13 (54.2)		
	College	5 (20.8)	4 (16.7)		
-Medical diseases	No	19 (79.2)	22 (91.7)	0.343	0.514
	DM	1 (4.2)	1 (4.2)		
	HT	2 (8.3)	1 (4.2)		
	RD	2 (8.3)	0 (0.0)		
-The presence of medical diseases	Yes	5 (20.8)	2 (8.3)	0.416	0.520
	No	19 (79.2)	22 (91.7)		
-Psychiatric Disorder	No	22 (91.7)	21 (87.5)	0.371	0.514
	AD	0 (0.0)	1 (4.2)		
	Depression	1 (4.2)	1 (8.3)		
	BD	1 (4.2)	0 (0.0)		
-Psychiatric disorder	Yes	2 (6.7)	3 (12.5)	>0.999	>0.999
	No	22 (91.7)	21 (87.5)		
Paternal Data					
-Educational Level	Elementary	11 (45.8)	10 (41.7)	0.739	0.839
	High School	10 (41.7)	9 (37.5)		
	College	3 (12.5)	5 (20.8)		
-Medical diseases	No	21 (87.5)	21 (87.5)	0.554	0.693
	DM	1 (4.2)	2 (8.3)		
	RD	3 (8.3)	1 (4.2)		
-Psychiatric disorder	No	24 (100.0)	22 (91.7)	0.113	0.227
	AD	0 (0.0)	2 (8.3)		

Fisher exact test, DM, diabetes mellitus; HT, hypertension; RD, rheumatological disease; BD, bipolar affective disorder; AD, anxiety disorder.

A history of developmental regression was reported in 62.5% of children with ASD (*n* = 11), while no cases were observed in the healthy control group (0.0%). This difference was found to be statistically significant (adjusted *p* = 0.009). In terms of speech development, 45.8% of children with ASD (*n* = 11) had no speech development, whereas all healthy controls (100%, *n* = 24) demonstrated normal speech development. This difference was also statistically significant (adjusted *p* = 0.009). Other variables including abortion history, cesarean delivery, prematurity, birth complications, neonatal incubator care, and presence of comorbid illness did not show statistically significant differences between groups (all *p* > 0.05) (Table 2).

**Table 2:** Birth history, developmental characteristics, and comorbidity status in children with autism spectrum disorder and healthy controls.

Variable		ASD	Control	*P* values	Adjusted *p*
		(*n* = 24)	(*n* = 24)		
		Counts (%)	Counts (%)		
Course of pregnancy	Normal	21 (87.5)	23 (95.8)	0.609	0.781
	Threat of miscarriage	3 (12.5)	1 (4.2)		
Cesarean delivery	Yes	15 (62.5)	17 (70.8)	0.561	0.757
	No	9 (37.5)	7 (29.2)		
History of pregnancy loss	Yes	6 (25.0)	8 (33.3)	0.525	0.738
	No	18 (75.0)	16 (66.7)		
Developmental regression	Yes	11 (47.8)	0 (0.0)	<0.001	0.009
	No	12 (52.2)	24 (100.0)		
Prematurity	Yes	2 (8.3)	4 (16.7)	0.666	0.832
	No	22 (91.7)	20 (83.3)		
Number of birth complications	No	20 (83.3)	20 (83.3)	0.178	0.400
	Hypoxia	2 (8.3)	0 (0.0)		
	Premature Birth	2 (8.3)	4 (16.7)		
First hour after birth	Normal	19 (79.2)	18 (75.0)	0.731	0.878
	Incubator Care	5 (20.8)	6 (25.0)		
Presence of speech	No	13 (54.2)	0 (0.0)	<0.001	0.009
	Yes	11 (45.8)	24 (100.0)		
Comorbid medical diseases	No	20 (83.3)	21 (87.5)	0.834	0.926
	Allergy	2 (8.3)	2 (8.3)		
	Epilepsy	2 (8.3)	1 (4.2)		

Fisher exact test.

A statistically significant difference was detected in the FXIII-Val34Leu polymorphism: 8 of the 24 children with ASD (33.3%) had the heterozygous FXIII-Val34Leu genotype, compared to 2 of 24 controls (8.3%), a difference that was statistically significant (*p* = 0.036) However, no statistically significant differences were observed between groups for the G20210A FII, FVL G1691A, C677T MTHFR, A1298C MTHFR or PAI-1 4G/5G polymorphisms (all *p* > 0.05) (see Table 3).

**Table 3:** Distribution of thrombophilia-related genetic polymorphisms in children with ASD and healthy controls.

Variable		Children Groups			
		ASD	Controls	Total	p^a^
		*N* = 24	*N* = 24	*n* = 48	
Genes	Genotypes	Counts (%)	Counts (%)	Counts (%)	
G20210A FII	GG (normal)	23 (95,8)	23 (95,8)	46 (95,8)	>0.999
	GA (heterozygous)	1 (4.2)	1 (4.2)	2 (4.2)	
	AA (mutant)	0 (0.0)	0 (0.0)	0 (0.0)	
FVL G1691A	GG (normal)	24 (100)	20 (83.3)	44 (71.7)	0.109
	GA (heterozygous)	0 (0)	4 (16.7)	4 (8.3)	
	AA (mutant)	0 (0.0)	0 (0.0)	0 (0.0)	
C677T MTHFR	C667 (normal)	12 (50.0)	15 (62.5)	27 (56.3)	0.655
	C667T(heterozygous)	10 (41.7)	8 (33.3)	18 (37.5)	
	667T (mutant)	2 (8.3)	1 (42.)	3 (6.3)	
A1298C MTHFR	AA (normal)	10 (41.7)	8 (33.3)	18 (37.5)	0.729
	AC (heterozygous)	10 (41.7)	13 (54.2)	23 (47.9)	
	CC (mutant)	4 (16.7)	3 (12.5)	7 (14.6)	
PAI 4G/5G	4G/4G	7 (29.2)	5 (20.8)	12 (25)	0.761
	4G/5G	9 (37.5)	8 (33.3)	17 (35.4)	
	5G/5G	8 (33.3)	11 (45.8)	19 (39.6)	
FXIII Val34Leu	VV (Normal)	15 (62.5)	22 (91.7)	37 (77.1)	0.036
	VL (heterozygous)	9 (37.5)	2 (8.3)	11 (22.9)	
	LL	0 (0.0)	0 (0.0)	0 (0.0)	

a Fisher's Exact Test.

There was no significant difference in the mean age between the ASD and control groups. (4.3 ± 1.0 vs. 4.7 ± 1.2 years, adjusted *p* = 0.317). The age at onset of speech was markedly delayed in the ASD group (35.8 ± 9.7 months) compared to the control group (20.5 ± 8.4 months), and this difference was statistically significant (adjusted *p* = 0.004) (see Table 4).

**Table 4:** Demographic features, developmental scale scores, and biochemical parameters of children with ASD and healthy controls.

Variable	ASD	Control	*P* values	Adjusted p
	(*n* = 24)	(*n* = 24)		
	Mean ± Sd.	Mean ± Sd.		
Age (years)	4.3 ± 1.0	4.7 ± 1.2	0.211	0.317
Birth weight (grams	3,198.1 ± 433.0	3,130.4 ± 417.4	0.672	0.873
Maternal age at admission (years)	32.8 ± 4.3	32.9 ± 6.4	0.772	0.908
Maternal age at childbirth (years)	28.5 ± 4.4	28.2 ± 6.1	0.926	0.962
Paternal age at diagnosis (years)	36.3 ± 7.1	35.5 ± 6.6	0.796	0.908
Paternal age at childbirth (years)	32.6 ± 7.7	30.9 ± 6.3	0.649	0.873
Breastfeeding (months)	13.8 ± 8.3	13.5 ± 8.9	0.875	0.962
Age at onset of speech (months)	35.8 ± 9.7	20.5 ± 8.4	<0.001	0.004
ADSI-language and cognitive	37.5 ± 9.5	48.0 ± 9.8	<0.001	0.004
ADSI-fine Motor	17.6 ± 2.5	21.3 ± 3.9	<0.001	0.004
ADSI -gross Motor	22.7 ± 3.9	22.5 ± 1.3	0.164	0.298
ADSI -social skills and self-care	29.4 ± 4.5	33.0 ± 6.7	0.002	0.013
ADSI-total score	107.1 ± 18.6	125.7 ± 17.7	<0.001	0.004
ABC-sensory score	10.2 ± 4.3	0	-	-
ABC-relating score	12.0 ± 7.1	0	-	-
ABC-body concept and object use	12.4 ± 7.0	0	-	-
ABC-language score	8.6 ± 6.2	0	-	-
ABC-social and self-help	7.1 ± 4.0	0	-	-
ABC-total score	50.4 ± 21.5	0	-	-
Vitamin B12 level (pg/mL)	386.7 ± 189.5	299.0 ± 119.9	0.183	0.302
Vitamin D level (ng/mL)	24.2 ± 8.6	26.6 ± 12.6	0.627	0.873
ASO titer (IU/mL)	57.6 ± 81.9	29.6 ± 14.8	0.192	0.308

Mann–Whitney, Student's *t*-test, ADSI, the ankara development screening inventory; ABC, autism behavior checklist; Sd., standard deviation; ASO, anti-streptolysin O.

The mean ABC score for children with ASD was 50.4 (±21.5). The ABC score for the control group was 0. All subdomains of the ADSI developmental assessment, except for the gross motor domain, yielded significantly lower scores in children diagnosed with ASD compared to healthy controls. Specifically, the language and cognitive subscale scores were markedly reduced in the ASD group (37.5 ± 9.5) relative to controls (48.0 ± 9.8; (adjusted *p* = 0.004). The total ADSI score was significantly reduced in the ASD group (107.1 ± 18.6) compared to healthy peers (125.7 ± 17.7; adjusted *p* = 0.004), indicating a generalized developmental delay in multiple domains among children with ASD. No statistically significant differences were observed in birth weight, parental ages, breastfeeding duration, or biochemical markers such as vitamin B12, vitamin D, and anti-streptolysin O (ASO) titers (all *p* > 0.05) (Table 4).

The frequencies of genetic polymorphisms were compared between mothers of children with ASD and control group mothers. A significant difference was found for the FXIII Val34Leu polymorphism. The heterozygous variant was more prevalent among mothers of children with ASD (54.2%) than control mothers (16.7%), while the normal genotype was more prevalent in the control group (83.3%) than among mothers of children with ASD (45.8%) (*p* = 0.015) (see Table 5 and Figure 2). However, no statistically significant differences were found between the ASD and control mothers for any of the other polymorphisms examined (FII G20210A, FVL G1691A, MTHFR C677T, MTHFR A1298C, or PAI-1 4G/5G; see Table 5).

**Table 5:** Distribution of thrombophilia-related genetic polymorphisms in mothers of children with ASD and mothers of healthy children.

Variable		Mothers of Children with ASD	Control Mothers	Total	*P* ^a^
		(*N* = 24)	(*N* = 24)		
Genes	Genotypes	Count (%)	Count (%)	Count (%)	
G20210A FII	GG (normal)	24 (100)	24 (100)	48 (100)	>0.999
	GA (heterozygous)	0 (0.0)	0 (0.0)	0 (0.0)	
	AA (mutant)	0 (0.0)	0 (0.0)	0 (0.0)	
FVL G1691A	GG (normal)	24 (100)	21 (87.5)	45 (93.8)	0.234
	GA (heterozygous)	0 (0.0)	3 (12.5)	3 (6.3)	
	AA (mutant)	0 (0.0)	0 (0.0)	0 (0.0)	
C677T MTHFR	C667 (normal)	8 (33.3)	11 (45.8)	19 (39.6)	0.556
	C667T(heterozygous)	16 (66.7)	13 (54.2)	29 (60.4)	
	667T (mutant)	0 (0.0)	0 (0.0)	0 (0.0)	
A1298C MTHFR	AA (normal)	9 (37.5)	9 (37.5)	18 (37.5)	>0.999
	AC (heterozygous)	12 (50.0)	13 (54.2)	25 (52.1)	
	CC (mutant)	3 (12.5)	2 (8.3)	5 (10.4)	
PAI 4G/5G	4G/4G	2 (8.3)	0 (0.0)	2 (4.2)	0.595
	4G/5G	15 (62.5)	16 (66.7)	31 (64.6)	
	5G/5G	7 (29.2)	8 (33.3)	15 (31.3)	
FXIII Val34Leu	VV (Normal)	11 (45.8)	20 (83.3)	31 (64.6)	0.015
	VL (heterozygous)	13 (54.2)	4 (16.7)	17 (35.4)	
	LL	0 (0.0)	0 (0.0)	0 (0.0)	

a Fisher's Exact Test.

Bias-reduced logistic regression analysis employing the Firth's method revealed a positive association between the FXIII Val34Leu heterozygous polymorphism and ASD in both mothers and children. In the parsimonious Firth logistic regression model, both child age and the combined maternal–child genotype variable (FXIII Val34Leu mother–child) were significant predictors. This variable was coded as “1” if either the mother or child carried the polymorphism, and as “0” if neither did. However, because the minor allele frequency was low and several contingency table cells had expected counts of less than five, modelling maternal and child genotypes as two separate predictors led to non-convergence, quasi-complete separation, and unstable estimates under limited events per parameter. To preserve identifiability and reduce variance inflation, a dyad-level indicator was specified (coded 1 if either the mother or the child carried the risk allele, and 0 otherwise; variable name: FXIII V34L mother-child). This specification was predefined as an exploratory, stability-oriented analytical choice and does not imply the biological equivalence of maternal and fetal genetic effects.

The presence of the FXIII Val34Leu mother-child polymorphism in either the mother or the child increased the odds of the outcome by a factor of approximately 4.130 [adjusted odds ratio (AOR) = 4.130, 95% confidence interval (CI) = 1.180–5.300, *p* = 0.027] (see Table 6). Although the confidence intervals were relatively wide due to the small sample size and low mutation frequency, the direction and magnitude of the effect were consistent with the hypothesized biological mechanism. Combining maternal and child genotypes into a single variable improved model stability and provided a more meaningful representation of familial genetic predisposition (see Table 6).

**Table 6:** Results of logistic regression analysis for variables associated with ASD risk.

Variable	B	SE	AOR	95% CI (AOR)		*P*
				Lower	Upper	
Child age	−0.620	0.280	0.540	0.300	0.890	0.021
FXIII-Val34Leu mother- child	1.420	0.650	4.130	1.180	5.300	0.027

AOR, adjusted odds ratio; Cl, confidence interval.

## Discussion

This pioneering study provides a detailed examination of a hereditary thrombophilia panel in children with ASD and their mothers, with a particular focus on the FXIII Val34Leu variant and its relationship to ASD. Significant differences were identified in children with ASD in terms of a history of developmental regression, delayed speech development and pronounced delays in the language and social domains during early childhood. From a genetic perspective, Factor XIII V34L polymorphism was significantly more prevalent in both children with ASD and their mothers. Furthermore, regression analysis revealed significant associations between the Factor XIII V34L mutation and ASD. These findings raise the question of whether hereditary thrombophilia contributes to ASD pathogenesis through intrauterine vascular mechanisms. Given the small sample size and pilot nature of our study, these results cannot be generalized. Therefore, further large-scale, longitudinal studies are required to investigate the relationship between thrombophilia markers and ASD.

In the etiopathogenesis of ASD, abnormalities in vasculogenesis and angiogenesis, structural alterations of the blood-brain barrier, and dysfunctions of the neurovascular unit have been emphasized (14). Particularly, genetic abnormalities affecting neuronal migration and signaling in the cerebral cortex during the intrauterine period are reported to trigger neurodevelopmental disorders (15, 16). In our study, we detected a significantly higher frequency of the FXIII Val34Leu polymorphism in children diagnosed with ASD and their mothers compared to healthy controls. This finding has not been reported in previous ASD literature, highlighting its significance and providing a new perspective on etiological mechanisms. Thrombophilia mutations are generally known to affect placental circulation and lead to pregnancy complications, thereby influencing fetal brain development (17). From this perspective, it seems likely that thrombotic events during the fetal period could lead to neuroinflammation. Moreover, vascular abnormalities in the perinatal period have been reported to increase the risk of ischemic and hemorrhagic stroke, resulting in neurodevelopmental problems (18, 19). Therefore, further studies are warranted to explore the role of thrombophilia parameters such as Factor XIII V34L in the development of ASD.

The FVL G1691A mutation is a common hereditary thrombophilia variant in the general population and is associated with an increased risk of thrombosis. This mutation may lead to complications such as venous thromboembolism (VTE) and small for gestational age (SGA) infants during pregnancy (20). In our study, the frequency of Factor V Leiden mutation was lower in both children with ASD and their mothers compared to the control group, and no statistically significant difference was observed.

The G20210A FII mutation is another important variant that increases the risk of thrombosis by elevating circulating prothrombin levels (21). In our cohort, this mutation was found at similarly low frequencies across groups. Among MTHFR enzyme mutations, the most common C677T variant is known to elevate plasma homocysteine levels and has been associated with preeclampsia, neural tube defects, and thrombotic events (22). Although experimental models and meta-analyses in the literature suggest a potential role of MTHFR variants in increasing ASD risk (23, 24), no significant differences were found in the distribution of C677T and A1298C MTHFR variants between the ASD and control groups in our study. These findings are consistent with those reported by Sener et al. (2014) and suggest that the role of these mutations in ASD risk warrants further investigation (25).

In our study, no statistically significant difference was found between the ASD and control groups in terms of PAI-1 4G/5G polymorphism. However, the literature indicates that the 4G/4G and 4G/5G genotypes of the PAI-1 gene are associated with thrombosis and early pregnancy complications, particularly with a significantly increased risk of miscarriage (26). Although the underlying mechanisms of the relationship between ASD and thrombophilia remain unclear, it has been proposed that hypercoagulability may lead to early spontaneous clot formation, triggering neuroinflammation and subsequently increasing autism risk (27). Recent studies have reported a positive correlation between hypercoagulability and neuroimmune parameters in children with ASD, particularly with elevated levels of antibodies against myelin basic protein (28). Furthermore, there is emerging evidence of associations between placental inflammation, maternal vascular pathology, and ASD risk. These findings support the hypothesis that ASD may be a neuroinflammatory disorder of vascular origin and underscore the role of cerebrovascular and immunological factors in its etiopathogenesis (29). The current body of evidence highlights the need for further comprehensive research into the pathophysiology of ASD.

The perinatal period is a critical phase characterized by a high risk of stroke and is associated with long-term neurological morbidities such as motor delay, cognitive impairments, speech disorders, and epilepsy (19). In pediatric populations, one of the major causes of hemorrhagic strokes is inherited coagulation disorders, particularly those related to factor deficiencies such as hemophilia (19). Both ischemic and hemorrhagic strokes during the perinatal period have been reported to increase the risk of neurodevelopmental disorders such as ASD and ADHD (18, 19, 30, 31). Notably, post-ischemic epilepsy is frequently observed, and ASD risk is significantly higher in children with comorbid epilepsy following ischemic stroke (30, 32). Although this was not directly evaluated in our study, these findings emphasize the need for a more detailed investigation into the relationship between ASD risk and neurovascular and thrombotic events.

It is established that hereditary thrombophilia mutations may trigger vascular complications during pregnancy via placental mechanisms, potentially leading to conditions such as fetal growth restriction (8). Notably, maternal FVL mutation may adversely affect fetal growth independently of placental pathology (8). Maternal metabolic and cardiovascular conditions (obesity, diabetes, underlying cardiac disease) increase the risk of preeclampsia (33, 34). Preeclampsia, in turn, can lead to severe complications such as fetal death, intrauterine growth restriction, and prematurity (33, 34). Furthermore, maternal metabolic syndrome, hypertension, and diabetes have been associated with an increased risk of thrombosis during pregnancy, which may impair fetal circulation and predispose to various neurodevelopmental disorders, including ASD (34). The literature also indicates that perinatal factors such as prematurity, preeclampsia, fetal distress, and cesarean delivery are associated with an increased risk of ASD (14, 35). In this context, our study supports further investigation into the role of perinatal and vascular factors in assessing the risk of ASD.

The primary limitations of our study are its pilot nature, cross-sectional design, multiple testing, sex imbalance, small sample size, and lack of investigation into complications such as perinatal stroke. Furthermore, this small-scale study did not group patients based on other mental health conditions or the severity of ASD. Key strengths of this study include the inclusion of both ASD children and their mothers (a family-based approach), the presence of a well-matched healthy control group, and a detailed, comprehensive analysis of an extended thrombophilia panel.

The study found that children diagnosed with ASD and their mothers had a higher frequency of the FXIII-Val34Leu polymorphism than healthy controls. Logistic regression analysis suggests this polymorphism may be a risk factor for ASD. Although no significant differences were observed between the groups regarding other thrombophilia-related genetic parameters, the high overall prevalence of certain thrombophilia polymorphisms among all children is noteworthy. In conclusion, our findings, representing the preliminary results of a potential link between the FXIII-Val34Leu mutation and ASD, are expected to contribute to the elucidation of underlying pathogenic mechanisms and should be supported by future molecular and clinical research.

## Data Availability

The datasets presented in this study can be found in online repositories. The names of the repository/repositories and accession number(s) can be found in the article/Supplementary Material.
